# Limited Utility of Plasma M30 in Discriminating Non-Alcoholic Steatohepatitis from Steatosis – A Comparison with Routine Biochemical Markers

**DOI:** 10.1371/journal.pone.0105903

**Published:** 2014-09-03

**Authors:** Wah-Kheong Chan, Pavai Sthaneshwar, Nik Raihan Nik Mustapha, Sanjiv Mahadeva

**Affiliations:** 1 Gastroenterology and Hepatology Unit, Gastrointestinal Endoscopy Unit, Department of Medicine, Faculty of Medicine, University of Malaya, Kuala Lumpur, Malaysia; 2 Division of Laboratory Medicine, Department of Pathology, Faculty of Medicine, University of Malaya, Kuala Lumpur, Malaysia; 3 Department of Pathology, Hospital Alor Setar, Alor Setar, Kedah, Malaysia; Bambino Gesu' Children Hospital, Italy

## Abstract

**Introduction:**

The utility of Cytokeratin-18 fragment, namely CK18Asp396 (M30), for the diagnosis of non-alcoholic steatohepatitis (NASH) is currently uncertain. We aimed to provide further data in this area among multi-ethnic Asian subjects with NAFLD.

**Materials and Methods:**

The accuracy of M30 for detecting NASH was compared with serum alanine aminotransferase (ALT), aspartate aminotransferase (AST) and gamma glutamyl transpeptidase (GGT) levels in consecutive adult subjects with biopsy-proven non-alcoholic fatty liver disease (NAFLD).

**Results:**

Data for 93 NAFLD subjects (mean age 51.0±11.1 years old and 51.6% males) and 20 healthy controls (mean age 50.2±16.4 years old and 33.3% males) were analyzed. There were 39 NASH subjects (41.9%) and 54 non-NASH subjects (58.1%) among the NAFLD subjects. Plasma M30 (349 U/L vs. 162 U/L), and serum ALT (70 IU/L vs. 26 IU/L), AST (41 IU/L vs. 20 IU/L) and GGT (75 IU/L vs. 33 IU/L) were significantly higher in NAFLD subjects than in healthy controls. Serum ALT (86 IU/L vs. 61 IU/L), AST (58 IU/L vs. 34 IU/L) and GGT (97 IU/L vs. 56 IU/L) were significantly higher in NASH subjects compared to non-NASH subjects, but no significant difference was observed with plasma M30 (435 U/L vs. 331 U/L). The accuracy of plasma M30, and serum ALT, AST and GGT was good for predicting NAFLD (AUROC 0.91, 0.95, 0.87 and 0.85, respectively) but less so for NASH (AUROC 0.59, 0.64, 0.75 and 0.68, respectively). Serum ALT and AST, but not plasma M30 showed a significant trend with increasing grades of ballooning and lobular inflammation.

**Conclusion:**

The utility of M30 in the detection of NASH in clinical practice appears limited, in comparison to routine biochemical markers.

## Introduction

The prevalence of non-alcoholic fatty liver disease (NAFLD) has increased rapidly over the years, parallel to the increase in metabolic syndrome, and it is recognized as one of the most common causes of chronic liver disease worldwide [1]. NAFLD encompasses a spectrum of liver conditions, ranging from simple steatosis to non-alcoholic steatohepatitis (NASH) to fibrosis and cirrhosis. While simple steatosis is generally considered benign, NASH may lead to fibrosis and eventually cirrhosis, with an increased risk of morbidity and mortality [2], [3].

The diagnosis of NASH is made by histopathological examination of a liver biopsy specimen. However, liver biopsy is invasive and it is associated with a small risk of serious complications [4]. It is not practical to subject all subjects with NAFLD to a liver biopsy to diagnose NASH. Furthermore, repeated liver biopsies to monitor disease progression in clinical practice is not acceptable either. A simple and reliable non-invasive test is needed for the diagnosis and follow-up of NASH.

Cytokeratin 18 (CK-18) is the major intermediate filament protein in liver cells and it is cleaved by caspases that are activated during apoptosis of liver cells, a process which plays an important role in NASH [5]. CK-18 fragment, namely CK18Asp396 (M30), has been studied for the diagnosis of NASH with varying results [6]–[13]. Whilst some studies have suggested that specific cut-off levels of CK-18 can reliably detect NASH in a cohort of NAFLD subjects [6]–[10], others have not shown such promising results [11]–[13]. These contrasting data may have been due to studies with a small sample size or from inadequate samples of cases with a range of NAFLD histology.

In an effort to provide more balanced data on the role of M30 in NAFLD, we have conducted a study in multi-ethnic Asian subjects with NAFLD. In this study, we aimed to evaluate the accuracy of plasma M30 in detecting NASH, and to compare it with some routine biochemical markers, namely serum alanine aminotransferase (ALT), aspartate aminotransferase (AST) and gamma glutamyl transpeptidase (GGT).

## Materials and Methods

Consecutive adult subjects (aged ≥18 years) with NAFLD who were scheduled for a liver biopsy were prospectively recruited between November 2012 and October 2013 for this study. The diagnosis of NAFLD was based on ultrasonography finding of fatty liver and exclusion of significant alcohol intake, use of medications that can cause fatty liver, viral hepatitis B and C infection, and other causes of chronic liver disease where indicated [14].

Demographic, anthropometric, relevant clinical and laboratory data were obtained using a standard protocol on the day of the liver biopsy procedure. Weight and height were measured using standard equipment. Body mass index (BMI) was calculated by dividing weight in kilogram by the square of height in meters. Waist circumference (WC) was measured at the mid-point between the lowest margin of the least palpable rib and the top of the iliac crest in the standing position. Blood pressure was measured in the sitting position using standardized equipment. A patient was considered hypertensive if there was a self-reported history of hypertension, if the patient was on anti-hypertensive medication(s), if the systolic blood pressure was ≥130 mmHg, or if the diastolic blood pressure was ≥85 mmHg.

All subjects had venous blood drawn after an overnight fast on the day of the liver biopsy procedure for complete blood count, blood glucose, glycated haemoglobin (HbA1c), lipid profile, liver profile, tests for viral hepatitis B and C infection, and for measurement of plasma M30 level. Biochemical measurements were performed using standard laboratory procedures. A patient was considered to have diabetes mellitus if there was a self-reported history of diabetes mellitus, if the patient was on anti-diabetic medication(s), or if fasting blood sugar was ≥7.0 mmol/L. A patient was considered to have dyslipidemia if there was a self-reported history of dyslipidemia, if the patient was on lipid-lowering medication(s), if the serum total cholesterol (TC) was ≥5.2 mmol/L, if the serum triglyceride (TG) was ≥1.7 mmol/L, if the serum high-density lipoprotein (HDL) was <1.0 mmol/L for men or <1.3 mmol/L for women, or if the serum low-density lipoprotein (LDL) was ≥3.4 mmol/L. Our laboratory's upper limit of normal for liver enzymes were as follow: alkaline phosphatase (ALP) 136 IU/L, aspartate aminotransferase (AST) 37 IU/L, alanine aminotransferase (ALT) 65 IU/L and gamma-glutamyl transpeptidase (GGT) 55 IU/L. The Elecsys HBsAg II assay and the Elecsys Anti-HCV II assay (Roche, Mannheim, Germany) were used to test for viral hepatitis B and C infection, respectively.

Controls were recruited from persons attending the Endoscopy Unit, University of Malaya Medical Centre for investigation of dyspepsia or screening colonoscopy. All controls had no history of chronic liver disease and had an ultrasound examination to exclude fatty liver. The presence of hypertension, diabetes mellitus and dyslipidemia was based on self-report. BMI and WC was determined as described above. Venous blood was drawn after an overnight fast for liver profile and for measurement of plasma M30 level.

### Measurement of plasma M30 level

The blood sample for measurement of plasma M30 level was collected in a plain tube on the same day of the liver biopsy procedure. The blood sample was processed to plasma and stored at −80°C until further analysis. The plasma was subsequently used for quantitative measurement of M30 using the M30-Apoptosense ELISA kit (PEVIVA, Bromma, Sweden). The test was performed for all samples in a single session by a single investigator (PS).

### Liver biopsy and histological assessment

Ultrasonography-guided percutaneous liver biopsy was performed by either one of two experienced operators (WKC, SM) using 18 G Temno ® II semi-automatic biopsy needle (Cardinal Health, Dublin, Ohio, USA). Liver biopsy specimens were processed using standard laboratory procedures. Liver biopsy slides were stained with hematoxylin and eosin stain and masson trichrome stain. Liver biopsy slides were examined by an experienced histopathologist (NRNM) who was blinded to clinical data. Histopathological findings were reported according to the Non-Alcoholic Steatohepatitis Clinical Research Network Scoring System [15]. The NAFLD activity score (NAS) is the sum of scores for hepatic steatosis (0–3), lobular inflammation (0–3) and hepatocyte ballooning (0–2). NAS 0–2 is not diagnostic of NASH, 3–4 is probable NASH and 5–8 is definite NASH. Subjects with NAS<5 was considered as non-NASH while subjects with NAS≥5 were considered to have NASH. Fibrosis was staged 0–4 (0 = no fibrosis, 1 = mild fibrosis, 2 = moderate fibrosis, 3 = severe fibrosis, 4 = cirrhosis).

### Ethics statement

This study was approved by the University of Malaya Medical Centre's Medical Ethics Committee and conformed to the Declaration of Helsinki. All subjects who participated provided written informed consent.

### Statistical analysis

Data were analysed using a standard statistical software program (SPSS 15.0). Continuous variables were expressed as mean ± standard deviation or median (interquartile range) and analyzed using t-test, ANOVA, Mann-Whitney test or Kruskal-Wallis test, as appropriate. Categorical variables were expressed as percentages and analyzed using chi-square test or Fisher's exact test, as appropriate. Significance was assumed when p<0.05. The performance of plasma M30 and serum ALT, AST and GGT levels for prediction of NAFLD and NASH was determined using area under receiver-operating characteristics curve (AUROC). AUROC was interpreted as follows: 0.90–1.00 = excellent, 0.80–0.90 = good, 0.70–0.80 = fair, <0.70 = poor. The sensitivity, specificity, positive predictive value and negative predictive value using cut-off values for high sensitivity, highest overall accuracy and high specificity were determined. Boxplots were used to compare the distribution of plasma M30 and serum ALT, AST and GGT levels across the different grades of steatosis, lobular inflammation and ballooning, and across the different stages of fibrosis.

## Results

### Patient characteristics

Ninety-three NAFLD subjects and 20 controls were recruited during the period of study. The characteristics of subjects are shown in Table 1. Controls and NAFLD subjects were well-matched in age and gender. NAFLD subjects had a greater BMI and WC and had higher prevalence of diabetes mellitus, hypertension and dyslipidemia compared to controls. There was a lower proportion of males among NASH subjects compared to non-NASH subjects. NASH and non-NASH subjects were similar in age, BMI, WC and prevalence of diabetes mellitus, hypertension and dyslipidemia. The quality of liver biopsy specimen as reflected by its length and the number of portal tracts, were also similar between NASH and non-NASH subjects. NASH subjects showed greater steatosis, lobular inflammation, ballooning and fibrosis.

**Table 1:** Patient characteristics.

	Controls, n = 20	NAFLD patients, n = 93	Non-NASH patients, n = 54	NASH patients, n = 39
Age, years	50.6±16.8	51.0±11.1	50.2±11.3	52.2±10.8
Male, %†	30.0	51.6	63.0	35.9
BMI, kg per m^2^ *	22.5±2.8	29.4±3.8	29.1±3.7	29.8±4.0
WC, cm*	81.8±7.6	97.7±9.7	96.8±9.7	98.8±9.7
Diabetes mellitus, %*	0	59.1	51.9	69.2
Hypertension, %*	20.0	88.2	85.2	92.3
Dyslipidemia, %*	40.0	96.8	94.4	100
Serum ALT, IU/L* †	26 (22–32)	70 (44–109)	61 (44–93)	86 (55–121)
Serum AST, IU/L* ‡	20 (18–27)	41 (28–64)	34 (25–46)	58 (38–78)
Serum GGT, IU/L* †	33 (22–45)	75 (47–125)	56 (40–101)	97 (53–151)
Plasma M30, U/L*	162 (103–215)	349 (257–612)	332 (249–534)	435 (279–758)
Liver biopsy length, mm	–	15.0±3.9	14.5±4.2	15.7±3.5
Number of portal tracts	–	8 (7–10)	8 (6–10)	9 (7–11)
Steatosis†				
0	–	3.2	5.6	0
1	–	34.4	42.6	23.1
2	–	47.3	48.1	46.2
3	–	15.1	3.7	30.8
Lobular inflammation‡				
0	–	4.3	7.4	0
1	–	53.8	81.5	15.4
2	–	38.7	11.1	76.9
3	–	3.2	0	7.7
Ballooning‡				
0	–	14.0	24.1	0
1	–	60.2	70.4	46.2
2	–	25.8	5.6	53.8
Fibrosis‡				
0	–	30.1	44.4	10.3
1	–	43.0	42.6	43.6
2	–	6.5	1.9	12.8
3	–	18.3	7.4	33.3
4	–	2.2	3.7	0

NAFLD, non-alcoholic fatty liver disease; NASH, non-alcoholic steatohepatitis; BMI, body mass index; WC, waist circumference; ALT, alanine aminotransferase; AST, aspartate aminotransferase; GGT, gamma glutamyl transpeptidase.

* Significant at p<0.001 between healthy controls and NAFLD patients.

† Significant at p<0.05.

‡ Significant at p<0.001, between non-NASH and NASH patients.

### Plasma M30 and serum ALT, AST and GGT levels in controls and NAFLD subjects

Plasma M30 levels were significantly higher in subjects with NAFLD (median 349 U/L, IQR 257 U/L–612 U/L) than in controls (median 162 U/L, IQR 103 U/L–215 U/L, p<0.001). Although plasma M30 levels were higher in subjects with NASH (median 435 U/L, IQR 279 U/L–758 U/L) compared to non-NASH subjects (median 332 U/L, IQR 249 U/L–534 U/L), the difference was not significant statistically (p = 0.145).

Serum ALT levels were significantly higher in subjects with NAFLD (median 70 IU/L, IQR 44 IU/L–109 IU/L) than in controls (median 26 IU/L, IQR 22 IU/L–32 IU/L, p<0.001). More importantly, serum ALT levels were significantly higher in NASH subjects (median 86 IU/L, IQR 55 IU/L–121 IU/L) compared to non-NASH subjects (median 61 IU/L, IQR 44 IU/L–93 IU/L, p<0.05).

Serum AST levels were significantly higher in subjects with NAFLD (median 41 IU/L, IQR 28 IU/L–64 IU/L) than in controls (median 20 IU/L, IQR 18 IU/L–27 IU/L, p<0.001). Serum AST levels were also significantly higher in NASH subjects (median 58 IU/L, IQR 38 IU/L–78 IU/L) compared to non-NASH subjects (median 34 IU/L, IQR 25 IU/L–46 IU/L, p<0.001).

Serum GGT levels were significantly higher in subjects with NAFLD (median 75 IU/L, IQR 47 IU/L–125 IU/L) than in controls (median 33 IU/L, IQR 22 IU/L–45 IU/L, p<0.001). Serum GGT levels were also significantly higher in NASH subjects (median 97 IU/L, IQR 53 IU/L–151 IU/L) compared to non-NASH subjects (median 56 IU/L, IQR 40 IU/L–101 IU/L, p<0.05).

### Prediction of NAFLD and NASH

**Figure 1:**
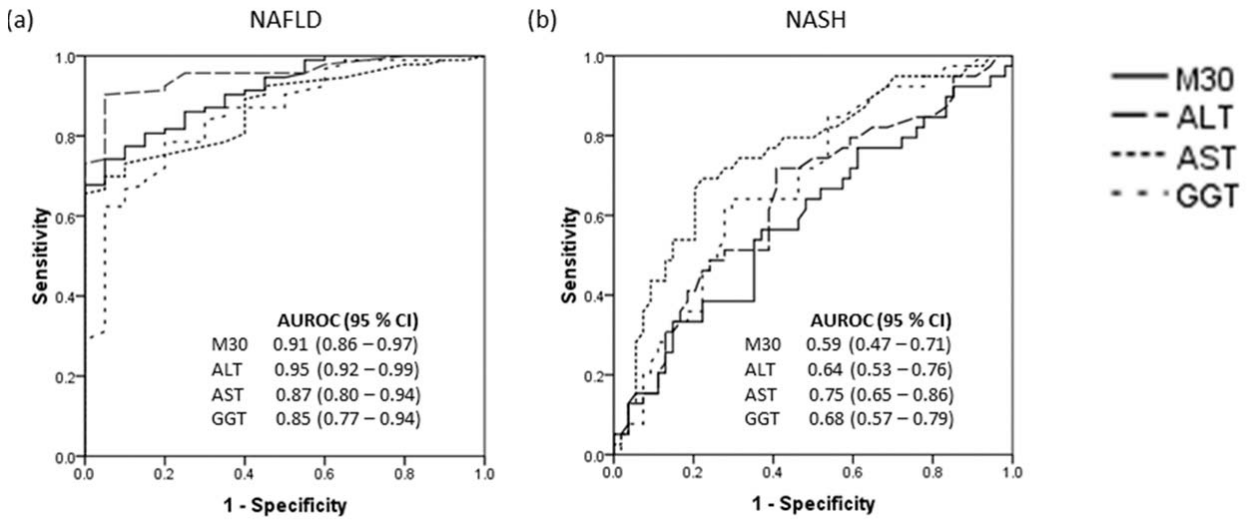
The receiver operating characteristic curves of plasma M30 and serum ALT, AST and GGT for prediction of (a) NAFLD, and (b) NASH. AUROC was interpreted as follows: 0.90–1.00 = excellent, 0.80–0.90 = good, 0.70–0.80 = fair, <0.70 = poor. ALT, alanine aminotransferase; AST, aspartate aminotransferase; GGT; gamma glutamyl transpeptidase; NAFLD, non-alcoholic fatty liver disease; NASH, non-alcoholic steatohepatitis.

The receiver operating characteristic curves of plasma M30 and serum ALT, AST and GGT for prediction of NAFLD and NASH are shown in Figure 1a–b. Plasma M30 and serum ALT levels were excellent for predicting NAFLD with an AUROC of 0.91 and 0.95, respectively. Serum AST and GGT levels were good for predicting NAFLD with an AUROC of 0.87 and 0.85, respectively. Serum AST level was fair for predicting NASH among NAFLD subjects with AUROC of 0.75. Plasma M30 and serum ALT and GGT levels were poor for predicting NASH among NAFLD subjects with an AUROC of 0.59, 0.64 and 0.68, respectively. The sensitivity, specificity, positive predictive value and negative predictive value when using the different cut-offs of plasma M30 and serum ALT, AST and GGT levels for prediction of NAFLD and NASH are shown in Tables 2 and 3.

**Table 2:** Accuracy of plasma M30 and serum ALT, AST and GGT for prediction of NAFLD.

	Cut-off, U/L or IU/L*	Sensitivity, %	Specificity, %	PPV, %	NPV, %
Plasma M30	162	95.7	45.0	89.0	69.2
	263	74.2	95.0	98.6	44.2
	278	67.7	100	100	40.0
Serum ALT	27	95.7	55.0	90.8	73.3
	35	90.3	95.0	98.8	67.9
	47	73.1	100	100	44.4
Serum AST	21	92.5	55.0	90.5	61.1
	33†	65.6	100	100	38.5
Serum GGT	32	90.3	50.0	89.4	52.6
	46	78.5	80.0	94.8	44.4
	55	62.4	95.0	98.3	35.2

ALT, alanine aminotransferase; AST, aspartate aminotransferase; GGT, gamma glutamyl transpeptidase; NAFLD, non-alcoholic fatty liver disease; PPV, positive predictive value; NPV, negative predictive value.

* Cut-off with high sensitivity, highest overall accuracy and high specificity were presented.

† Cut-off for highest overall accuracy and high specificity were the same.

**Table 3:** Accuracy of plasma M30 and serum ALT, AST and GGT for prediction of NASH.

	Cut-off, U/L or IU/L*	Sensitivity, %	Specificity, %	PPV, %	NPV, %
Plasma M30	293	71.8	40.7	46.7	66.7
	432	56.4	63.0	52.4	66.7
	474	43.6	64.8	47.2	61.4
Serum ALT	53	79.5	40.7	49.2	73.3
	67	71.8	59.3	56.0	74.4
	100	41.0	79.6	59.3	65.2
Serum AST	30	84.6	40.7	50.8	78.6
	48	69.2	77.8	69.2	77.8
	65	43.6	90.7	77.3	69.0
Serum GGT	49	84.6	42.6	51.6	79.3
	84	64.1	70.4	61.0	73.1
	109	46.2	77.8	60.0	66.7

ALT, alanine aminotransferase; AST, aspartate aminotransferase; GGT, gamma glutamyl transpeptidase; NASH, non-alcoholic steatohepatitis; PPV, positive predictive value; NPV, negative predictive value.

* Cut-off with high sensitivity, highest overall accuracy and high specificity were presented.

When global histological assessment was used for the diagnosis of NASH, 44 patients who were considered as non-NASH using the NAS had NASH. The characteristics of patients with and without NASH and the receiver operating characteristic curves of plasma M30 and serum ALT, AST and GGT for prediction of NASH when global histological assessment was used can be found as supplementary material (Table S1 and Figure S1, respectively). The findings were similar regardless of whether NAS or global histological assessment was used for the diagnosis of NASH.

### Plasma M30 and serum ALT, AST and GGT according to steatosis, ballooning, lobular inflammation and fibrosis

Plasma M30 and serum ALT and AST levels did not show any significant trend when analyzed according to steatosis grades. Although serum GGT level showed a significant trend when analyzed according to steatosis grades, the difference in serum GGT level was only significant between subjects with grade 2 and 3 steatosis (Figure S2).

Serum ALT and AST levels showed a significant trend with increasing grades of ballooning. However, this was not seen with plasma M30 and serum GGT levels. Serum ALT levels were significantly higher in subjects with grade 1 compared to subjects without ballooning. However, serum ALT levels were not significantly different between subjects with grade 1 and grade 2 ballooning. Serum AST levels were significantly higher in subjects with grade 2 compared to grade 1 ballooning and in subjects with grade 1 compared to subjects without ballooning (Figure S3).

Serum ALT and AST levels also showed a significant trend with increasing grades of lobular inflammation. Once again, this was not seen with plasma M30 and serum GGT levels. Serum ALT and AST levels were significantly higher in subjects with grade 2 compared to grade 1 lobular inflammation. However, serum ALT and AST levels were not significantly different between subjects with grade 2 and grade 3 lobular inflammation, and between subjects with grade 1 and subjects without lobular inflammation (Figure S4).

**Figure 2:**
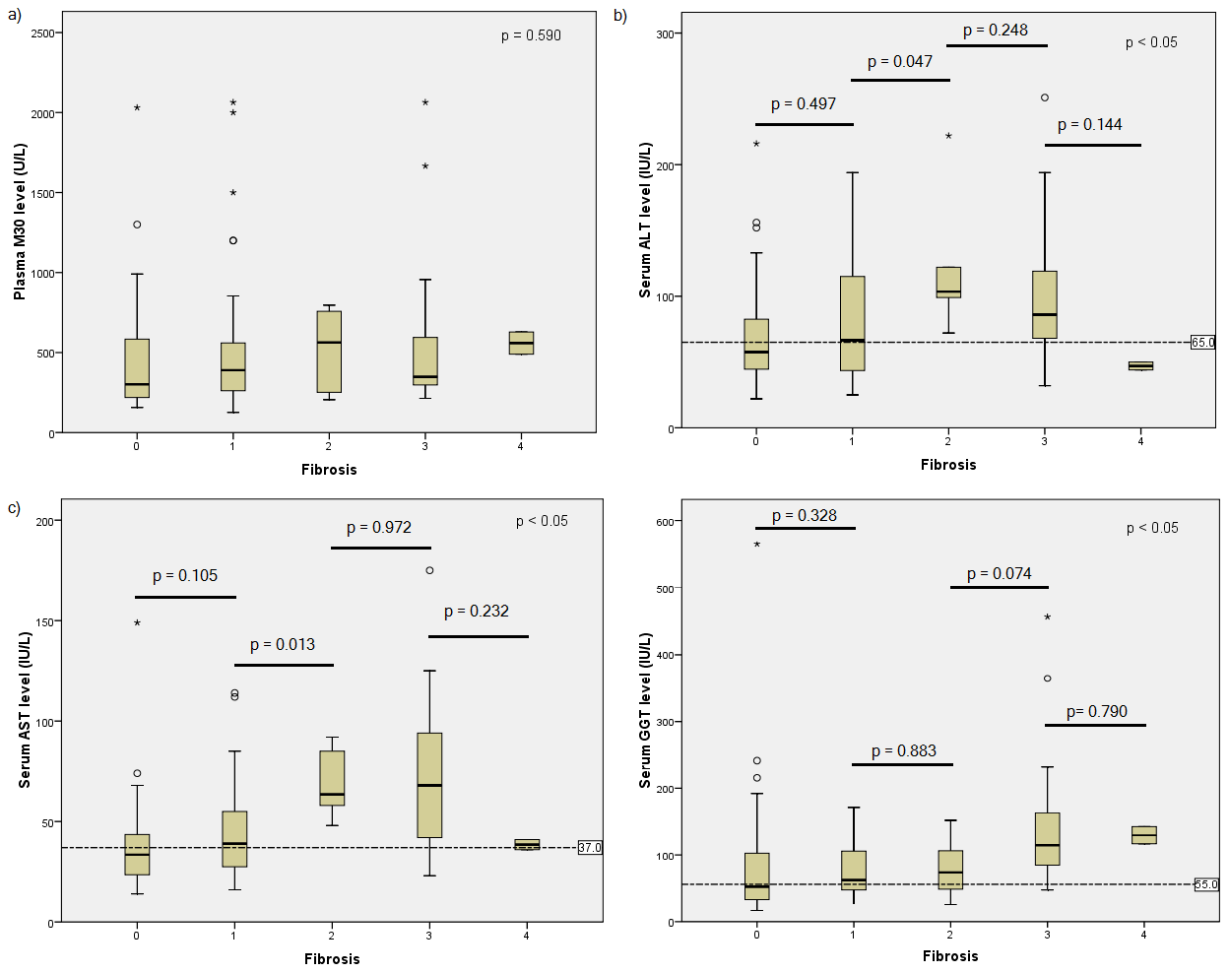
Plasma M30 and serum ALT, AST and GGT levels according to fibrosis stages. The data between and across groups were analyzed using Mann-Whitney test and Kruskal-Wallis test, respectively. The p value between groups were only shown when there was a significant difference across groups. Fibrosis was staged 0–4 (0 = no fibrosis, 1 = mild fibrosis, 2 = moderate fibrosis, 3 = severe fibrosis, 4 = cirrhosis). F, fibrosis stage; ALT, alanine aminotransferase; AST, aspartate aminotransferase; GGT; gamma glutamyl transpeptidase.

Plasma M30 level did not show any significant trend when analyzed according to fibrosis stages. There was a significant difference in serum ALT, AST and GGT levels across fibrosis stages. However, only differences in serum ALT and AST for stage 1 and stage 2 fibrosis was significant (Figure 2).

### Prediction of ballooning and lobular inflammation

**Figure 3:**
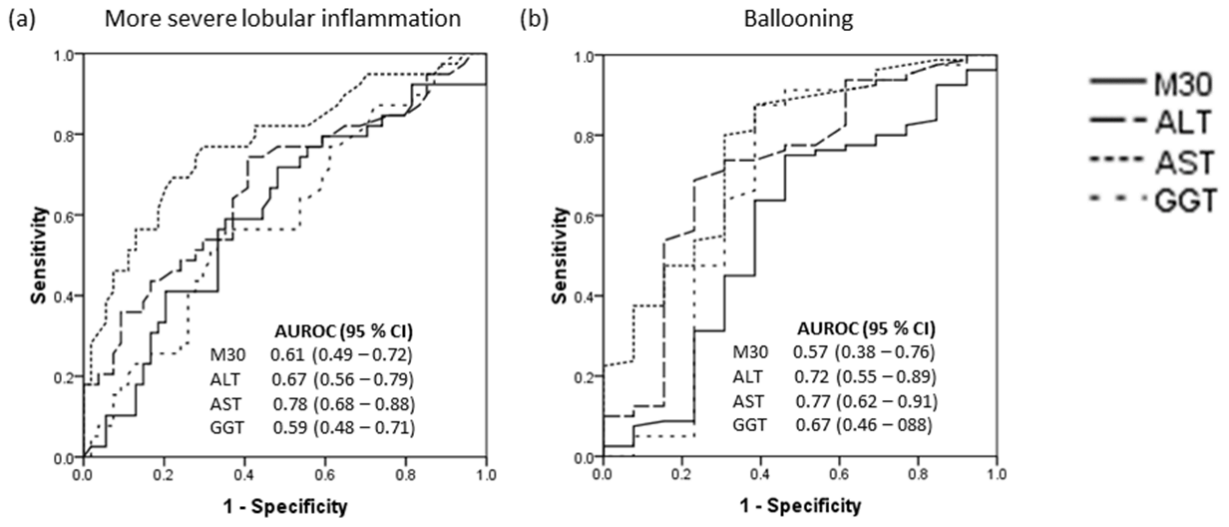
The receiver operating characteristic curves of plasma M30 and serum ALT, AST and GGT for prediction of (a) more severe lobular inflammation, and (b) ballooning. AUROC was interpreted as follows: 0.90–1.00 = excellent, 0.80–0.90 = good, 0.70–0.80 = fair, <0.70 = poor. ALT, alanine aminotransferase; AST, aspartate aminotransferase; GGT; gamma glutamyl transpeptidase.

In view of the above findings, analysis was carried out to determine the accuracy of plasma M30 and serum ALT, AST and GGT levels for prediction of presence of ballooning and presence of more severe lobular inflammation. Lobular inflammation grade 0 and grade 1 were considered less severe while grade 2 and grade 3 were considered more severe. The results are shown in Figure 3a–b. Serum ALT and AST levels were fair for prediction of presence of ballooning with AUROC of 0.72 and 0.77, respectively. Serum AST level was fair for prediction of presence of more severe lobular inflammation with AUROC of 0.78. Plasma M30 and serum GGT levels were poor for prediction of presence of ballooning and presence of more severe lobular inflammation. The sensitivity, specificity, positive predictive value and negative predictive value when using the different cut-offs of plasma M30 and serum ALT, AST and GGT levels for prediction of presence of ballooning and more severe lobular inflammation can be found as supplementary material (Table S2 and S3).

### Characteristics of patients according to plasma M30 tertiles and factors associated with plasma M30 level

The characteristics of patients according to plasma M30 tertiles can be found as supplementary material (Table S4). The only variables that showed significant difference across tertiles were serum ALT and GGT. On bivariate correlation, plasma M30 level was significantly correlated with BMI (r = 0.23, p<0.05), and serum ALT (r = 0.32, p<0.01) and AST (r = 0.35, p<0.01). Age, BMI, and serum ALT, AST and GGT were entered into multiple linear regression analysis but none of the variables were found to be significant (data not shown).

## Discussion

The potential use of plasma M30 level as a non-invasive biomarker to determine histological disease severity in NAFLD subjects was first reported by Wieckowska et al. In their study of 44 consecutive subjects with suspected NAFLD at the time of liver biopsy, plasma CK-18 levels were markedly increased in adults with NASH compared to those with simple steatosis or normal liver biopsies. Plasma M30 level had a high accuracy in distinguishing subjects with NASH from those with simple steatosis or normal liver biopsies with an AUROC of 0.93. Two subjects with borderline NASH were not included in the analysis [6]. In a subsequent multi-centre validation study consisting of 139 subjects, Feldstein et al demonstrated that plasma M30 level had an AUROC of 0.83 in detecting NASH. However, it is important to note that this population consisted of a relatively small percentage of subjects with borderline NASH (19%) [7]. Subsequently, Shen et al reported that M30 had an AUROC of 0.66 for detecting NASH in a study population which consisted of a larger percentage of subjects with borderline NASH (49.7%) [12]. In our study population which consisted of a similar proportion of subjects with borderline NASH (52.7%), we similarly demonstrated that plasma M30 was less useful for distinguishing NAFLD subjects with NASH from those without NASH with an AUROC of 0.59. In a recently published study consisting of 318 subjects, Cusi et al similarly reported that plasma M30 level was less useful for NASH diagnosis with an AUROC of 0.65 [13].

Although M30 appeared to have a limited value for detecting NASH in this study, we found that serum AST level had a reasonable accuracy in distinguishing NASH from non-NASH subjects with an AUROC of 0.75. Furthermore, serum AST level had a greater accuracy in predicting the presence of ballooning and the presence of more severe lobular inflammation in contrast to serum ALT and GGT levels. Other tests for the diagnosis of NASH such as measurement of total cell death markers M65 and M65ED, adipocyte fatty acid binding protein (AFABP) and fibroblast growth factors 21 (FGF21) have been studied but were not better with AUROC of 0.71, 0.70, 0.59 and 0.62, respectively [11], [12].

It appears that an accurate non-invasive test for NASH remains elusive. However, we should not forget that NASH is a continuous spectrum and markers may be variably expressed in each individual so that finding a test that confirms the presence or absence of NASH using a pre-determined cut-off may be difficult if not impossible. It may be more realistic to aim for a test that would reflect changes in severity of NASH when followed over time. For example, Suzuki et al reported that the combination of a baseline and rate of change of serum ALT and AST levels had an AUROC of 0.72 and 0.73, respectively, in predicting improvement, and an AUROC of 0.75 and 0.77, respectively, in predicting worsening of histological inflammation in NASH subjects. The AUROC improved to 0.88 and 0.89, respectively, when baseline histology was taken into consideration [16]. In another study of 36 subjects without NASH at baseline, Shen et al showed that changes in M30 was quite accurate in predicting the development of NASH with an AUROC of 0.82. Using 35 U/L as the cut-off for increment in M30, development of NASH could be predicted with a sensitivity and specificity of 80.0% and 81.5%, respectively [12]. The use of changes in plasma M30 and serum ALT and AST levels to predict changes in histology, particularly inflammation and ballooning, should be compared and deserves further studies in a larger group of subjects. A combination of various parameters, including diabetes mellitus, gender, BMI, triglycerides, M30 and M65–M30 has been proposed for non-invasive diagnosis of NASH [17]. Separately, a support vector machine-based panel using osteoprotegerin, fibroblast growth factor 21 and M30 also appeared promising for this purpose [18]. However, these requires further study and validation.

Our study was carried out prospectively and collection of blood samples were done on the same day as the liver biopsy procedure to minimize differences due to changes over time. Despite our best effort, the study had several limitations. Firstly, as in any study using histopathological examination of liver biopsy specimen as a reference, there may have been sampling and observer variability. Secondly, the absence of NAFLD in controls was based on ultrasonography, which may lack sensitivity in detection of mild hepatic steatosis. However, performing a liver biopsy to exclude NAFLD in these subjects was not deemed to be ethical in this study.

In conclusion, plasma M30 appears to have a limited utility in detecting NASH in clinical practice, particularly when compared to serum ALT, AST or GGT levels subjects. While other more accurate yet non-invasive tests are needed for the diagnosis of NASH, the use of changes in plasma M30 and serum ALT and AST levels to predict changes in histology, particularly inflammation and ballooning, should be compared and deserves further study in a larger group of subjects.

## Supporting Information

Figure S1 **The receiver operating characteristic curves of plasma M30 and serum ALT, AST and GGT for prediction of NASH when global histological assessment was used for the diagnosis of NASH.** AUROC was interpreted as follows: 0.90–1.00 = excellent, 0.80–0.90 = good, 0.70–0.80 = fair, <0.70 = poor. ALT, alanine aminotransferase; AST, aspartate aminotransferase; GGT; gamma glutamyl transpeptidase; NASH, non-alcoholic steatohepatitis.
(TIF)

Figure S2 **Plasma M30 and serum ALT, AST and GGT levels according to steatosis grades.** The data between and across groups were analyzed using Mann-Whitney test and Kruskal-Wallis test, respectively. The p value between groups were only shown when there was a significant difference across groups. Steatosis was graded 0–3 (0 = less than 5%, 1 = 5–33%, 2 = 34–66%, 3 = more than 66%). ALT, alanine aminotransferase; AST, aspartate aminotransferase; GGT; gamma glutamyl transpeptidase.
(TIF)

Figure S3 **Plasma M30 and serum ALT, AST and GGT levels according to ballooning grades.** The data between and across groups were analyzed using Mann-Whitney test and Kruskal-Wallis test, respectively. The p value between groups were only shown when there was a significant difference across groups. Ballooning was graded 0–2 (0 = none, 1 = few/mild, 2 = many/prominent). ALT, alanine aminotransferase; AST, aspartate aminotransferase; GGT; gamma glutamyl transpeptidase.
(TIF)

Figure S4 **Plasma M30 and serum ALT, AST and GGT levels according to lobular inflammation grades.** The data between and across groups were analyzed using Mann-Whitney test and Kruskal-Wallis test, respectively. The p value between groups were only shown when there was a significant difference across groups. Lobular inflammation was graded 0–3 (0 = none, 1 = less than 2 foci, 2 = 2–4 foci, 3 = more than 4 foci) ALT, alanine aminotransferase; AST, aspartate aminotransferase; GGT; gamma glutamyl transpeptidase.
(TIF)

Table S1 **Patient characteristics (diagnosis of NASH based on global histological assessment).**
(DOCX)

Table S2 **The sensitivity, specificity, positive predictive value and negative predictive value when using the different cut-offs of plasma M30 and serum ALT, AST and GGT levels for prediction of presence of more severe lobular inflammation.**
(DOCX)

Table S3 **The sensitivity, specificity, positive predictive value and negative predictive value when using the different cut-offs of plasma M30 and serum ALT, AST and GGT levels for prediction of presence of ballooning.**
(DOCX)

Table S4 **Characteristics of patients according to plasma M30 tertiles.**
(DOCX)

Data S1 **This file contains data underlying the findings described in this manuscript.**
(SAV)
