# Supplementary figures and images for: Limited Utility of Plasma M30 in Discriminating Non-Alcoholic Steatohepatitis from Steatosis – A Comparison with Routine Biochemical Markers
Source: PLoS One. 2014 Sep 3;9(9):e105903. doi: 10.1371/journal.pone.0105903 (PMC4153577; doi:10.1371/journal.pone.0105903)

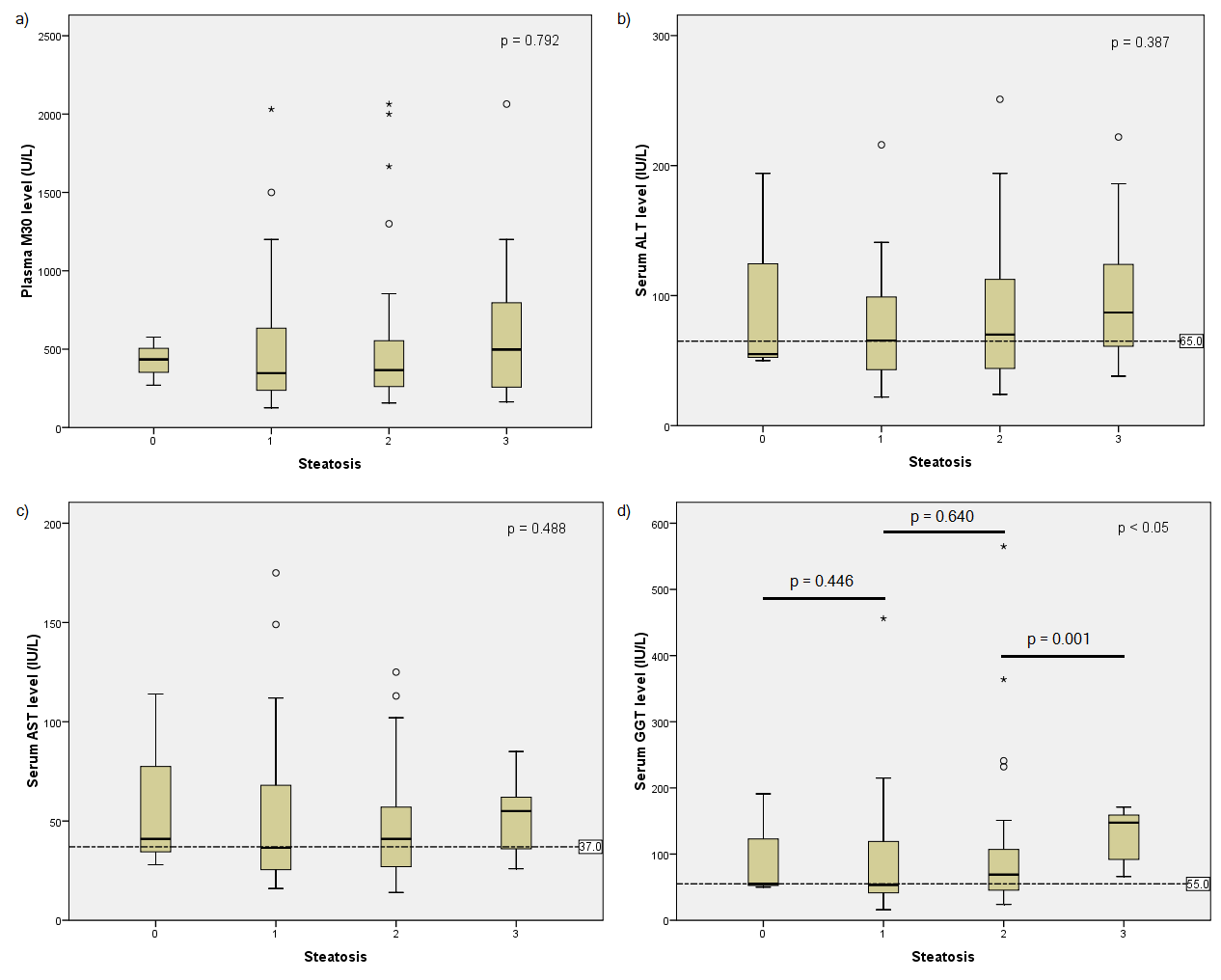

Supplement: Figure S2 — Plasma M30 and serum ALT, AST and GGT levels according to steatosis grades. The data between and across groups were analyzed using Mann-Whitney test and Kruskal-Wallis test, respectively. The p value between groups were only shown when there was a significant difference across groups. Steatosis was graded 0–3 (0 = less than 5%, 1 = 5–33%, 2 = 34–66%, 3 = more than 66%). ALT, alanine aminotransferase; AST, aspartate aminotransferase; GGT; gamma glutamyl transpeptidase. (TIF) [file pone.0105903.s002.tif]

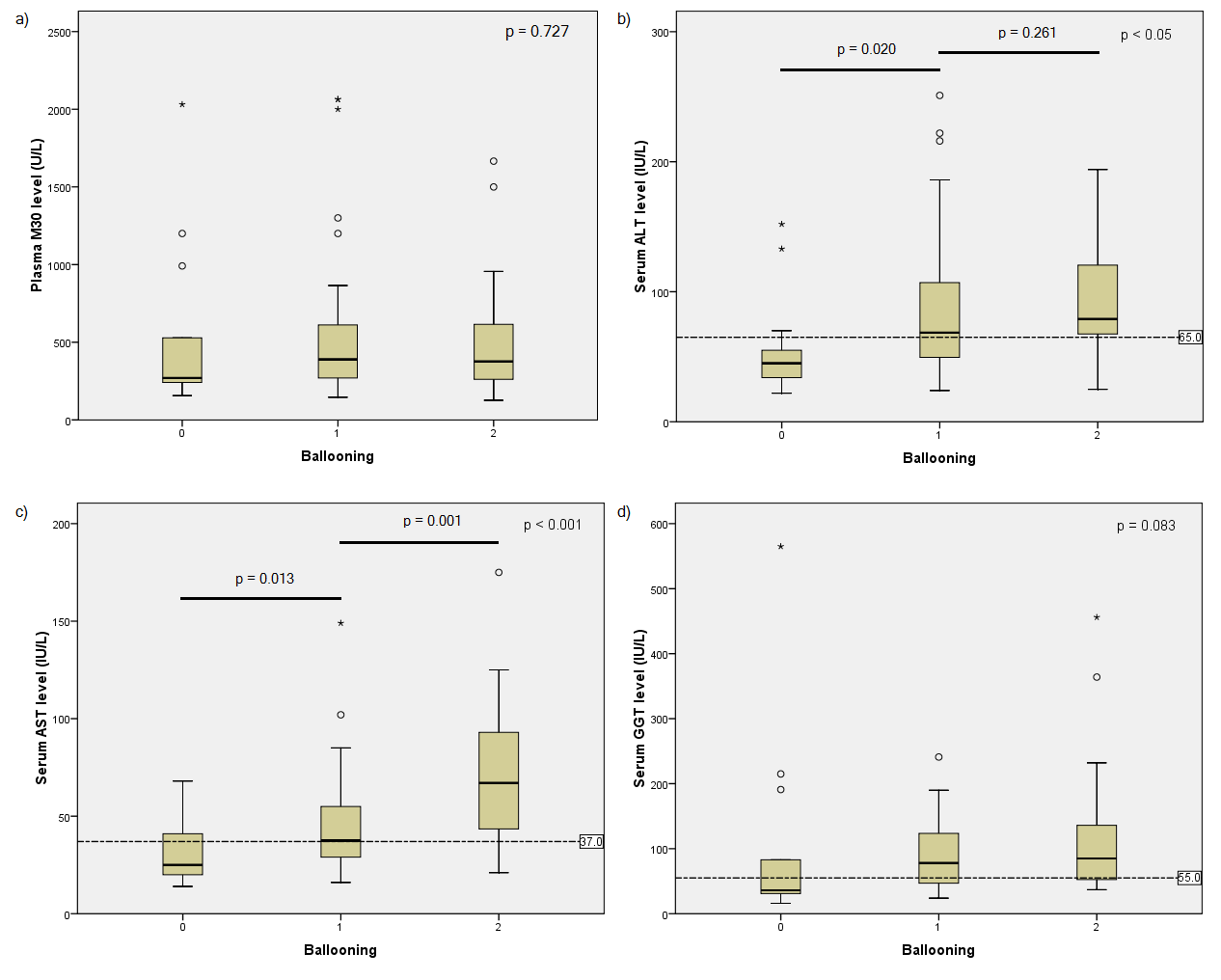

Supplement: Figure S3 — Plasma M30 and serum ALT, AST and GGT levels according to ballooning grades. The data between and across groups were analyzed using Mann-Whitney test and Kruskal-Wallis test, respectively. The p value between groups were only shown when there was a significant difference across groups. Ballooning was graded 0–2 (0 = none, 1 = few/mild, 2 = many/prominent). ALT, alanine aminotransferase; AST, aspartate aminotransferase; GGT; gamma glutamyl transpeptidase. (TIF) [file pone.0105903.s003.tif]

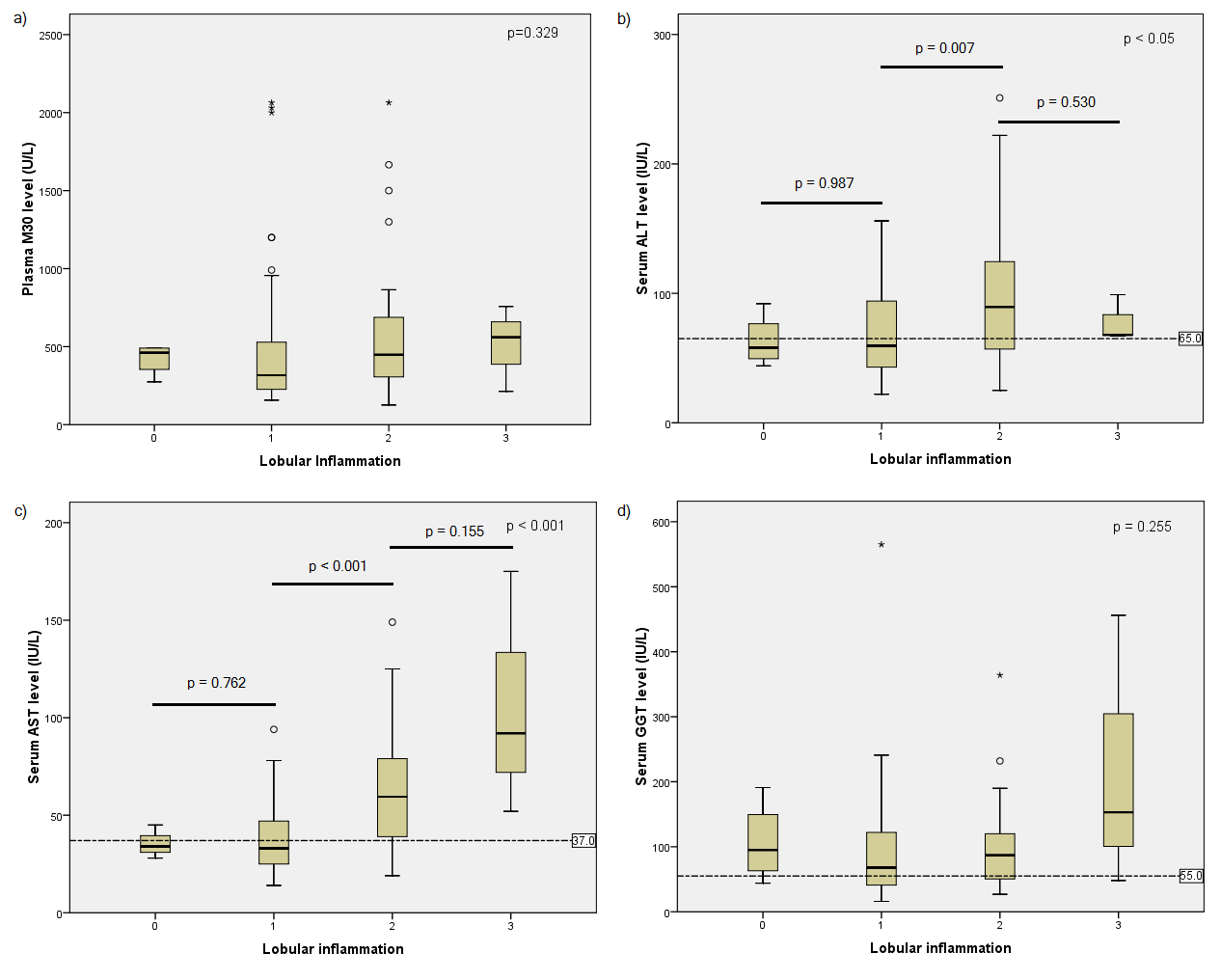

Supplement: Figure S4 — Plasma M30 and serum ALT, AST and GGT levels according to lobular inflammation grades. The data between and across groups were analyzed using Mann-Whitney test and Kruskal-Wallis test, respectively. The p value between groups were only shown when there was a significant difference across groups. Lobular inflammation was graded 0–3 (0 = none, 1 = less than 2 foci, 2 = 2–4 foci, 3 = more than 4 foci) ALT, alanine aminotransferase; AST, aspartate aminotransferase; GGT; gamma glutamyl transpeptidase. (TIF) [file pone.0105903.s004.tif]
