# Supplementary material for: Limited Utility of Plasma M30 in Discriminating Non-Alcoholic Steatohepatitis from Steatosis – A Comparison with Routine Biochemical Markers
Source: PLoS One. 2014 Sep 3;9(9):e105903. doi: 10.1371/journal.pone.0105903 (PMC4153577; doi:10.1371/journal.pone.0105903)
Supplement: Table S1 — Patient characteristics (diagnosis of NASH based on global histological assessment). (DOCX) [file pone.0105903.s005.docx]

**Table S1** Patient characteristics (diagnosis of NASH based on global histological assessment)

|  | Non-NASH patients, n = 10 | NASH patients, n = 83 |
| --- | --- | --- |
| Age, years | 46.9 ± 9.4 | 51.5 ± 11.2 |
| Male, % | 60.0 | 50.6 |
| BMI, kg per m^2^ * | 26.6 ± 2.7 | 29.7 ± 3.8 |
| WC, cm | 92.4 ± 6.3 | 98.3 ± 9.8 |
| Diabetes mellitus, % | 30.0 | 59.0 |
| Hypertension, % | 60.0 | 65.1 |
| Dyslipidemia, % | 90.0 | 79.5 |
| Serum ALT, IU/L | 51 (33 – 86) | 71 (44 – 110) |
| Serum AST, IU/L * | 27 (19 – 48) | 42 (29 – 65) |
| Serum GGT, IU/L | 48 (33 – 197) | 76 (47 – 124) |
| Plasma M30, U/L | 292 (204 – 644) | 387 (267 – 629) |
| Liver biopsy length, mm | 13.2 ± 2.5 | 15.3 ± 4.0 |
| Number of portal tracts * | 7 (6 – 8) | 9 (7 – 11) |
| Steatosis *  0  1  2  3 | 20.0  20.0  60.0  0 | 1.2  36.1  45.8  16.9 |
| Lobular inflammation *  0  1  2  3 | 10.0  90.0  0  0 | 3.6  49.4  43.4  3.6 |
| Ballooning †  0  1  2 | 100.0  0  0 | 3.6  67.5  28.9 |
| Fibrosis †  0  1  2  3  4 | 100.0  0  0  0  0 | 21.7  48.2  7.2  20.5  2.4 |

NASH, non-alcoholic steatohepatitis; BMI, body mass index; WC, waist circumference; ALT, alanine aminotransferase; AST, aspartate aminotransferase; GGT, gamma glutamyl transpeptidase

* Significant at p < 0.05

† Significant at p < 0.001, between non-NASH and NASH patients
