# Supplementary material for: Limited Utility of Plasma M30 in Discriminating Non-Alcoholic Steatohepatitis from Steatosis – A Comparison with Routine Biochemical Markers
Source: PLoS One. 2014 Sep 3;9(9):e105903. doi: 10.1371/journal.pone.0105903 (PMC4153577; doi:10.1371/journal.pone.0105903)
Supplement: Table S2 — The sensitivity, specificity, positive predictive value and negative predictive value when using the different cut-offs of plasma M30 and serum ALT, AST and GGT levels for prediction of presence of more severe lobular inflammation. (DOCX) [file pone.0105903.s006.docx]

**Table S2** Accuracy of plasma M30 and serum ALT, AST and GGT for prediction of presence of more severe lobular inflammation *

|  | Cut-off, U/L or IU/L † | Sensitivity, % | Specificity, % | PPV, % | NPV, % |
| --- | --- | --- | --- | --- | --- |
| Plasma M30 | 277 | 79.5 | 40.7 | 49.2 | 73.3 |
|  | 432 | 59.0 | 64.8 | 54.8 | 68.6 |
|  | 560 | 38.5 | 79.6 | 57.7 | 64.2 |
| Serum ALT | 53 | 79.5 | 40.7 | 49.2 | 73.3 |
|  | 66 | 74.4 | 59.3 | 56.9 | 76.2 |
|  | 109 | 38.5 | 85.2 | 65.2 | 65.7 |
| Serum AST | 30 | 84.6 | 40.7 | 50.8 | 78.6 |
|  | 42 | 76.9 | 70.4 | 65.2 | 80.9 |
|  | 69 | 38.5 | 94.4 | 83.3 | 68.0 |
| Serum GGT | 53 | 69.2 | 40.7 | 45.8 | 64.7 |
|  | 84 | 56.4 | 64.8 | 53.7 | 67.3 |
|  | 111 | 38.5 | 74.1 | 51.7 | 62.5 |

ALT, alanine aminotransferase; AST, aspartate aminotransferase; GGT, gamma glutamyl transpeptidase; NAFLD, non-alcoholic fatty liver disease; AUROC, area under receiver-operating characteristics curve; PPV, positive predictive value; NPV, negative predictive value

* Lobular inflammation grade 0 and 1 were considered less severe while grade 2 and 3 were considered more severe

† Cut-off with high sensitivity, highest overall accuracy and high specificity were presented
