# Supplementary material for: Limited Utility of Plasma M30 in Discriminating Non-Alcoholic Steatohepatitis from Steatosis – A Comparison with Routine Biochemical Markers
Source: PLoS One. 2014 Sep 3;9(9):e105903. doi: 10.1371/journal.pone.0105903 (PMC4153577; doi:10.1371/journal.pone.0105903)
Supplement: Table S3 — The sensitivity, specificity, positive predictive value and negative predictive value when using the different cut-offs of plasma M30 and serum ALT, AST and GGT levels for prediction of presence of ballooning. (DOCX) [file pone.0105903.s007.docx]

**Table S3** Accuracy of plasma M30 and serum ALT, AST and GGT for prediction of presence of ballooning

|  | Cut-off, U/L or IU/L * | Sensitivity, % | Specificity, % | PPV, % | NPV, % |
| --- | --- | --- | --- | --- | --- |
| Plasma M30 | 268 | 76.3 | 46.2 | 89.7 | 24.0 |
|  | 317 | 63.8 | 61.5 | 91.1 | 21.6 |
|  | 474 | 40.0 | 69.2 | 88.9 | 15.8 |
| Serum ALT | 45 | 77.5 | 46.2 | 89.9 | 25.0 |
|  | 57 | 68.8 | 76.9 | 94.8 | 28.6 |
|  | 89 | 40.0 | 84.6 | 94.1 | 18.6 |
| Serum AST | 25 | 90.0 | 46.2 | 91.1 | 42.9 |
|  | 29 | 80.0 | 69.2 | 94.1 | 36.0 |
|  | 51 | 40.0 | 84.6 | 94.1 | 18.6 |
| Serum GGT | 37 | 91.2 | 53.8 | 92.4 | 50.0 |
|  | 42 | 87.5 | 61.5 | 93.3 | 44.4 |
|  | 95 | 38.8 | 76.9 | 91.2 | 16.9 |

ALT, alanine aminotransferase; AST, aspartate aminotransferase; GGT, gamma glutamyl transpeptidase; NAFLD, non-alcoholic fatty liver disease; AUROC, area under receiver-operating characteristics curve; PPV, positive predictive value; NPV, negative predictive value

* Cut-off with high sensitivity, highest overall accuracy and high specificity were presented
