# Supplementary material for: Limited Utility of Plasma M30 in Discriminating Non-Alcoholic Steatohepatitis from Steatosis – A Comparison with Routine Biochemical Markers
Source: PLoS One. 2014 Sep 3;9(9):e105903. doi: 10.1371/journal.pone.0105903 (PMC4153577; doi:10.1371/journal.pone.0105903)
Supplement: Table S4 — Characteristics of patients according to plasma M30 tertiles. (DOCX) [file pone.0105903.s008.docx]

**Table S4** Characteristics of patients according to plasma M30 tertiles

|  | M30 tertiles | | |
| --- | --- | --- | --- |
|  | I | II | III |
| Age, years | 56.1 ± 11.4 | 48.4 ± 12.1 | 51.6 ± 9.5 |
| Male, % | 45.2 | 61.3 | 48.4 |
| Body mass index, kg per m^2^ | 28.2 ± 4.3 | 30.1 ± 3.4 | 29.8 ± 3.5 |
| Waist circumference, cm | 96.6 ± 11.8 | 29.8 ± 3.5 | 97.0 ± 7.5 |
| Diabetes mellitus, % | 58.1 | 54.8 | 54.8 |
| Hypertension, % | 67.7 | 61.3 | 64.5 |
| Dyslipidemia, % | 80.6 | 80.6 | 80.6 |
| Fasting blood sugar, mmol/L | 5.6 (5.0 – 6.3) | 5.6 (4.8 – 7.3) | 5.6 (4.9 – 8.1) |
| HbA1c, % | 6.90 (5.78 – 8.01) | 6.15 (5.60 – 7.43) | 6.30 (5.63 – 8.07) |
| Triglycerides, mmol/L | 1.7 ± 0.8 | 1.7 ± 0.5 | 1.7 ± 0.7 |
| Total cholesterol, mmol/L | 4.7 ± 1.2 | 5.0 ± 1.1 | 5.1 ± 1.3 |
| HDL, mmol/L | - 1. ± 0.2 | 1.1 ± 0.2 | 1.1 ± 0.2 |
| LDL, mmol/L | 2.9 ± 1.1 | 3.1 ± 1.0 | 3.2 ± 1.1 |
| ALP, IU/L | 74 (60 – 98) | 79 (66 – 90) | 82 (63 – 96) |
| ALT, IU/L * | 55 (35 – 72) | 72 (52 – 108) | 99 (50 – 125) |
| AST, IU/L | 29 (24 – 53) | 41 (31 – 54) | 60 (33 – 80) |
| GGT, IU/L * | 52 (36 – 92) | 86 (55 – 128) | 82 (44 – 151) |
| Steatosis  0  1  2  3 | 3.2  35.5  48.4  12.9 | 3.2  38.7  48.4  9.7 | 3.2  29.0  45.2  22.6 |
| Lobular inflammation  0  1  2  3 | 3.2  67.7  25.8  3.2 | 6.5  48.4  45.2  0 | 3.2  45.2  45.2  6.5 |
| Ballooning  0  1  2 | 22.6  54.8  22.6 | 6.5  64.5  29.0 | 12.9  61.3  25.8 |
| Fibrosis  0  1  2  3  4 | 41.9  38.7  6.5  12.9  0 | 19.4  48.4  3.2  25.8  3.2 | 29.0  41.9  9.7  16.1  3.2 |

* Significant at p < 0.05 across tertiles
